# Hospitalizations for Acute Otitis and Sinusitis in Patients Living with HIV: A Retrospective Analysis of a Tertiary Center in Romania

**DOI:** 10.3390/jcm13113346

**Published:** 2024-06-06

**Authors:** Vlad Ștefan Pleșca, Victor Daniel Miron, Adrian Gabriel Marinescu, Anca Cristina Drăgănescu, Anca Doina Pleșca, Oana Săndulescu, Cătălina Voiosu, Răzvan Hainăroșie, Anca Streinu-Cercel

**Affiliations:** 1 Carol Davila University of Medicine and Pharmacy, 050474 Bucharest, Romaniamironvictordaniel@gmail.com (V.D.M.);; 2 National Institute of Infectious Diseases “Prof. Dr. Matei Balș”, 021105 Bucharest, Romania; 3 Academy of Romanian Scientists (AOSR), 050044 Bucharest, Romania

**Keywords:** HIV, acute sinusitis, acute otitis, hospitalization

## Abstract

**Background/Objectives:** Acute or chronic ear, nose and throat (ENT) conditions in people living with HIV can lead to hospitalization and affect their quality of life. The aim of our study was to determine the frequency and characteristics of hospitalizations for acute sinusitis (AS) and acute otitis (AO) in people living with HIV. **Methods:** We performed a retrospective analysis over the course of six years (from January 2018 to December 2023), assessing all hospitalizations for AS and/or AO occurring in patients living with HIV, at the largest infectious diseases hospital in Romania. **Results:** We identified a total of 179 cases, among which 149 cases (83.2%) were attributed to AS and 41 cases (22.9%) were due to AO. Among cases of AS, maxillary sinuses were most frequently involved (*n* = 140/149, 94.0%), and among cases of AO, acute congestive otitis media (*n* = 14, 34.1%) and acute purulent otitis media (*n* = 13, 31.7%) were the most common forms. The underlying HIV infection was classified as stage C3 in 57.5% of cases. In 19.6% of cases, it was possible to identify either the trigger or the etiological agent, and the most frequent bacterial pathogens were *Streptococcus pneumoniae*, *Staphylococcus aureus*, *Haemophilus influenzae* and *Pseudomonas aeruginosa*. **Conclusions:** In conclusion, this study highlights that hospitalizations due to acute sinus and ear involvement are not isolated events in people living with HIV. A prospective follow-up is needed to gain a deeper and more dynamic understanding of how ENT health is affected in people with HIV infection. Furthermore, promoting prevention through vaccination may reduce to a certain extent the burden of ENT infections in this population.

## 1. Introduction

Forty years after its first documentation, HIV infection continues to represent a particular challenge for the global community, with considerable individual and public health impact. Despite significant improvement in prevention and treatment of HIV infection, certain challenges still remain, an important number of new cases are diagnosed each year [1], and many patients are hospitalized for AIDS-defining events as well as non-AIDS-defining events or other causes [2,3,4].

Published data show that a large share of people living with HIV experience ear, nose and throat (ENT) manifestations at some point during the course of their HIV infection and most often during the periods of immune suppression [5]. Although the clinical spectrum of ENT manifestations is very varied, sinus [5] and otic [6] involvement are among the most common. Sinusitis and otitis have been shown to occur more frequently in patients with infection who also have a CD4+ T-cell level below 200 cells/μL. Furthermore, patients with prolonged immunodeficiency display a higher tendency toward chronicity [7].

In Romania, the impact of ENT conditions in patients living with HIV has not yet been thoroughly quantified, despite several studies showing that various other chronic conditions are identified with a high frequency, which contributes to the complexity of the medical management of this patient cohort [8]. In addition, many of these individuals require hospitalization either due to worsening of chronic diseases or in response to various acute infectious episodes. This highlights the pressing need for a more integrated approach to the care of these patients, including increased attention to the prompt diagnosis and treatment of ENT conditions, which can significantly influence patients’ quality of life and their pathway through the medical care system. A better understanding of how ENT conditions affect patients with HIV could lead to improved prevention and treatment strategies, thereby reducing the frequency and severity of hospitalizations.

In this context, the aim of this analysis is to assess the frequency and characteristics of hospitalizations for acute sinusitis (AS) and acute otitis (AO) among people living with HIV, evaluated in the largest infectious diseases hospital in Romania.

## 2. Methods

We conducted a retrospective analysis among patients living with HIV who were hospitalized for AO or AS between January 2018 and December 2023 in the National Institute of Infectious Diseases “Prof. Dr. Matei Balș” (NIID) in Bucharest, Romania. NIID is the largest tertiary infectious disease center in the country and serves as the primary monitoring center for patients living with HIV in the southern region of Romania, as well as the coordinator of the national HIV network. NIID’s records currently include over 3000 patients diagnosed with HIV infection.

We included in the analysis all patients living with HIV who were hospitalized in NIID during the study period for acute illness (onset of symptoms not more than 7 days prior to hospitalization) and who were diagnosed with AO and/or AS by ENT assessment. Patients hospitalized for other acute conditions or those who presented with chronic otitis or sinusitis that did not necessitate hospitalization due to exacerbation were omitted from this study. Additionally, individuals who did not undergo an ENT assessment during their hospital stay were excluded, and so were patients whose medical records contained incomplete data as defined below.

Demographic data (such as sex and age), clinical data (signs and symptoms and duration of symptoms), laboratory data (CD4 level, blood count, inflammation markers and microbiology results), HIV stage, existence of antiretroviral treatment and evolution during hospitalization were collected for each patient. The ENT assessments in the patient records were checked by one of the ENT physicians in the study team to ensure correct classification of otic or sinus conditions. Otitis cases were classified into four categories: acute otitis externa (AOE), acute congestive otitis media (ACOM), acute otitis media with effusion (AOME) and acute purulent otitis media (APOM). Sinusitis cases were classified into acute maxillary sinusitis (AMS), acute ethmoid sinusitis (AES), acute frontal sinusitis (AFS) and acute sphenoid sinusitis (ASS).

The patients in this study were grouped into three age categories: children (ages below 18 years old), adults (18–65 years old) and elderly (over 65 years old). All the blood test result values outside the normal range as established by the laboratory were interpreted as either elevated or decreased, depending on the case, and were assessed for clinical relevance. HIV infection status was determined according to the Centers for Disease Control and Prevention classification [9].

Statistical analysis was performed using IBM SPSS Statistics software (version 25, IBM Corp., Armonk, NY, USA). A *p*-value < 0.05 was considered to be statistically significant. For categorical variables, we report the frequency (*n*) and percentage (%), and comparisons were performed using the Chi-square test. For continuous variables, after checking the normality distribution of the data with the Shapiro–Wilk test, we report the median and interquartile range (IQR, 25th and 75th percentiles), and comparisons were made using the Mann–Whitney U (U) and Kruskal–Wallis H (H) tests.

## 3. Results

### 3.1. General Characteristics

**Figure 1:**
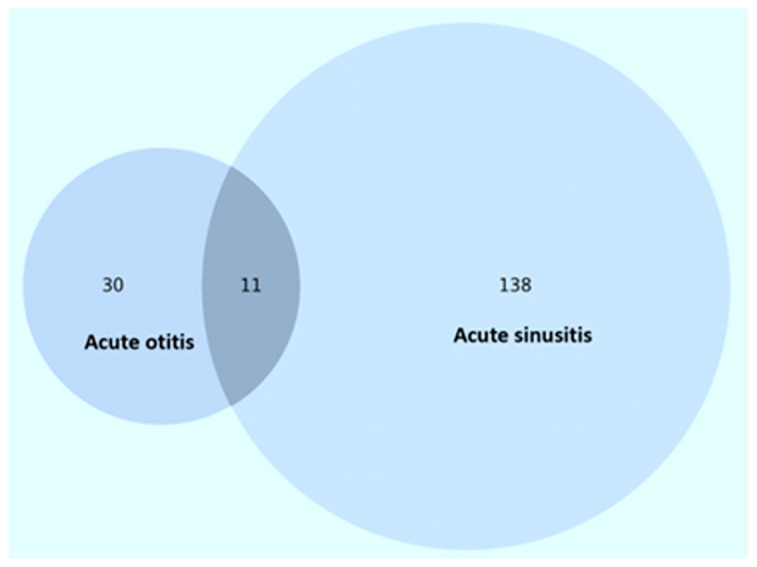
Case distribution of patients in the study.

During the 6-year study period, a total of 179 patients living with HIV were hospitalized for otitis and/or sinusitis. AS (*n* = 149, 83.2%) was the most common, occurring in 149 patients (83.2%), while AO was diagnosed in 41 patients (22.9%), and in 11 of these cases there was both otic and sinus involvement (Figure 1). There was a higher proportion of female patients (*n* = 104, 58.1%) relative to males (*n* = 75, 41.9%), and the median age was 32.5 years (IQR: 29.5, 42.3 years). Overall, adult patients (*n* = 159, 88.8%) predominated, while children (*n* = 16, 8.9%) and the elderly (*n* = 4, 2.2%) accounted for a low proportion.

**Figure 2:**
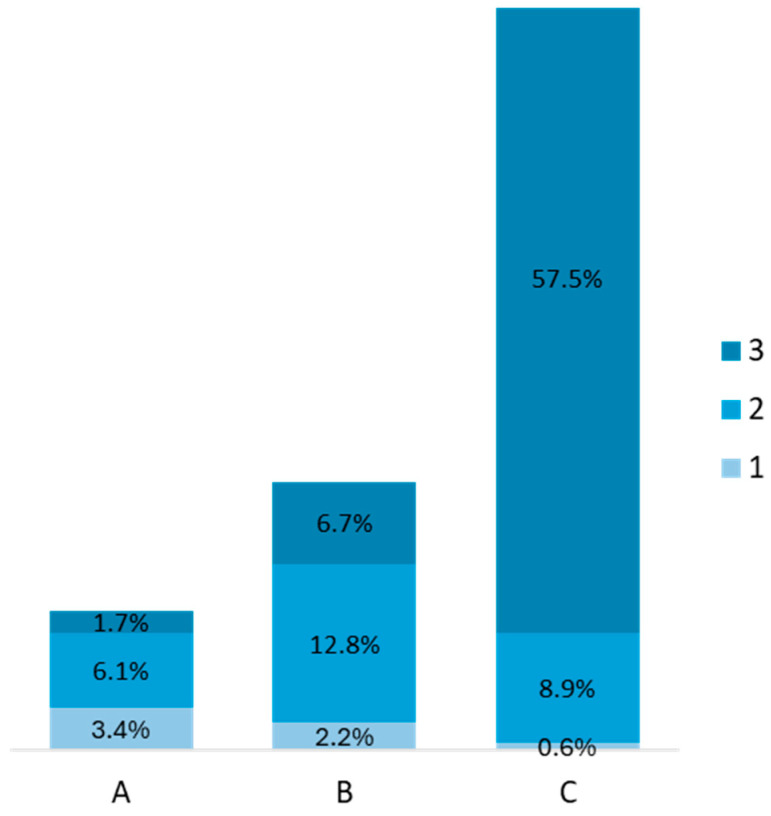
HIV infection stage of patients in the study (A, B, C—are the HIV stages).

The majority of patients with HIV infection were classified as stage C3 (*n* = 103, 57.5%), followed by stages B2 (*n* = 23, 12.8%) and C2 (*n* = 16, 8.9%), Figure 2. Median CD4 values at admission were 508 cells/mm^3^ (IQR: 246, 761 cells/mm^3^), and 95.5% of the patients (*n* = 171) were under antiretroviral treatment. HIV viral load was available in a total of 73 (40.8%) patients. Of these, 39.7% (*n* = 29) were undetectable, 26.0% (*n* = 19) had HIV RNA detected but <200 copies/mL, 9.6% (*n* = 7) had HIV RNA between 200 and 1000 copies/mL and 24.7% (*n* = 18) had HIV RNA >1000 copies/mL. Also, 39.7% (*n* = 71) had at least one associated chronic condition, the most common being chronic hepatitis B (*n* = 23, 12.8%), cardiovascular disease (*n* = 21, 11.7%), a recent history of or current tuberculosis (*n* = 13, 7.3%), obesity (*n* = 10, 5.6%) and neurological disease (*n* = 9, 5.0%).

### 3.2. Classification and Etiology of AS and AO

**Figure 3:**
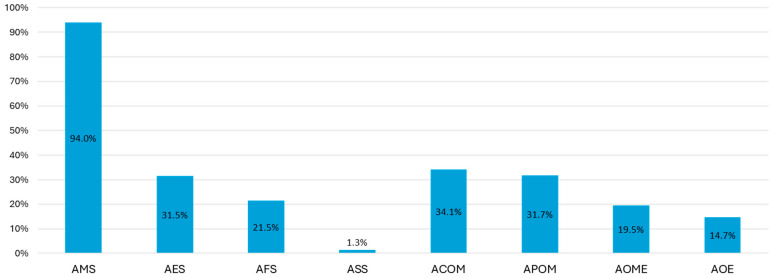
Frequency of types of acute sinusitis and otitis. The percentages have been calculated by taking as the denominator the total number of cases of AS and AO, as shown in the text. AMS—acute maxillary sinusitis, AES—acute ethmoid sinusitis, AFS—acute frontal sinusitis, ASS—acute sphenoid sinusitis, ACOM—acute congestive otitis media, APOM—acute purulent otitis media, AOME—acute otitis media with effusion, and AOE—acute otitis externa.

Of the 149 AS cases, maxillary sinus involvement was the most common (*n* = 140, 94.0%), followed by ethmoid sinus involvement (*n* = 47, 31.5%), Figure 3. In 62 cases (41.6%), the involvement of at least two different sinuses was identified.

Of the 41 patients with AO, 14 (34.1%) were diagnosed with ACOM and 13 (31.7%) with APOM, Figure 3. In the majority of cases, the otic involvement was unilateral (35/41, 85.4%).

Patient sex, HIV infection status or the presence of at least one chronic condition were not significantly associated with any of the sinus or otitis locations (*p* > 0.05 in each case). Instead, we identified that the median age of patients with AMS was significantly lower than those with other types of AS (32.9 years (IQR: 29.9, 41.5 years) vs. 43.7 years (IQR: 31.5, 48.4), *p* = 0.043, z = −2.020 and U = 376.5). Similarly, ACOM was identified in younger patients (28.2 years (IQR: 1.4, 30.6 years) vs. 32.6 years (IQR: 29.2, 48.9 years), *p* = 0.007, z = −2.681 and U = 106. 5), while AOE was identified in patients with an older median age compared to those with other types of AO (49.5 years (IQR: 35.5, 50.4 years) vs. 30 years (IQR: 3.8, 32.6 years), *p* = 0.001, z = −3.007 and U = 23.5).

In 35 cases (19.6%), it was possible to identify either the trigger or the etiological agent involved in the development of AS or AO. Influenza (*n* = 16, 8.9%) was the most common infection preceding the ENT involvement. Among the bacterial agents, *Streptococcus pneumoniae*, *Staphylococcus aureus*, *Haemophilus influenzae* and *Pseudomonas aeruginosa* were identified (Table 1). In 15 cases of AMS (15/140, 10.7%), the origin was identified as odontogenic.

### 3.3. Clinical and Laboratory Characteristics

There were no significant differences in the frequency of AO or AS in relation to the CD4 level or antiretroviral treatment status (Table 2). The clinical picture was dominated by fever (*n* = 118, 65.9%), nasal congestion (*n* = 114, 63.7%) and headache (*n* = 105, 58.7) as shown in Table 2. Patients with AS more frequently displayed symptoms such as nasal congestion (*p* < 0.001) or headache (*p* = 0.001) and had an underlying septum deviation (*p* = 0.018) or turbinate hypertrophy (*p* = 0.023), while patients with AO most frequently displayed otalgia as the presenting complaint (*p* < 0.001).

Increased white blood cell counts were seen in 50.8% of patients, especially in those with AO (*p* = 0.009, Table 2). Increased C-reactive protein values were observed in 74.3% of the cases, also with a higher frequency in patients with AO (83.3%) or those with both otitis and sinusitis (81.8%).

**Figure 4:**
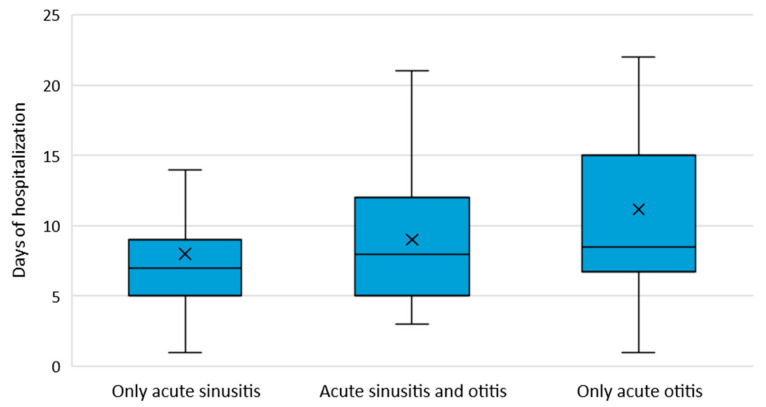
Median length of hospitalization by type of otic or sinus involvement.

Median length of hospital stay was 7 days (IQR: 5, 10 days), significantly higher in patients with AO (8.5 days (IQR: 7, 15 days)) compared to those with AS (7 days (IQR: 5, 9 days)), *p* = 0.016 and H = 8.318, Figure 4. Complications such as tympanic perforation (*n* = 12 cases), meningitis (*n* = 2 cases), cavernous sinus thrombosis (*n* = 1 case) and mastoiditis (*n* = 1 case) were documented in 8.9% (*n* = 16) of cases.

## 4. Discussion

In this study, we analyzed data from more than 3000 HIV-infected patients in the NIID registry over a six-year period. During this time, we identified 179 hospitalizations caused by AS and OA. All patients included in this study had been previously diagnosed with HIV infection. Our data show a 3.6-fold higher incidence of sinus involvement (149 cases of AS) compared to otic involvement (41 cases of AO). The majority of cases were identified among patients with stage C3 HIV infection, characterized by marked immunosuppression. This highlights the increased vulnerability of patients with a weakened immune system to ENT infections, which can rapidly progress to severe forms and the necessity of hospitalization. This phenomenon is particularly relevant as it indicates that ENT infections occur more frequently in advanced stages of HIV infection, highlighting the importance of careful monitoring and early interventions to prevent and manage these conditions. Thus, our results strongly suggest the necessity for a proactive and comprehensive approach to the screening and treatment of ENT infections in people living with HIV. This is particularly critical for those in advanced stages of the disease, where the immune system is significantly compromised. Implementing regular and thorough screening protocols can help detect ENT infections early, thus enabling timely and effective treatment. By doing so, we can prevent these infections from progressing to more severe forms that often require hospitalization. Additionally, early intervention and consistent management can substantially improve the quality of life for patients with HIV infection, minimizing discomfort and complications associated with ENT infections. Ultimately, this approach not only aims to enhance patient well-being but also to reduce the overall incidence of hospitalizations, thereby alleviating the burden on healthcare systems and improving long-term health outcomes for those living with HIV.

We identified that most cases were found in female patients, who represented 58.1% of all hospitalizations analyzed. This finding is consistent with the results of the study published by Shija et al. [5], which showed a 67.5% female predominance in ENT pathologies. This distribution reflects the global demographic ratios of HIV infection, where according to the Joint United Nations Programme on HIV and AIDS (UNAIDS), in 2022, more than 53% of people living with HIV globally were female [10], and this is particularly true for Romania as well, by contrast with most Central and Western European countries. Recent trends indicate an increase in new HIV cases [10], which could change epidemiological dynamics and influence the long-term distribution of HIV-related pathologies, including ENT. These observations highlight the importance of continuous monitoring of demographic trends of patients with HIV infection in order to optimize health resources and interventions.

Among patients with AS, maxillary sinus involvement was the most common (94.0%). Similarly, Miziara et al. indicated that the maxillary sinus was involved in allrhinosinusitis cases they analyzed [11]. Other older studies confirm the same trend, highlighting the high frequency of maxillary sinus involvement in the context of sinus infections [12,13]. It is important to note that in 41.6% of the cases, at least two sinuses located in different areas were involved, indicating the widespread nature of sinus infections among HIV patients. This statistic underscores the extensive reach these infections can have within the sinuses, often affecting multiple regions simultaneously. As a result, a complex and thorough ENT evaluation becomes essential. This evaluation typically includes the use of advanced diagnostic tools such as fibroscopy and various imaging techniques. Fibroscopy allows for a detailed visual inspection of the nasal passages and sinuses, while diagnostic imaging, including computer tomography scans or magnetic resonance imaging, can provide comprehensive views of the sinus cavities. These methods are crucial for accurately characterizing the full extent of sinus involvement, which may not be apparent through basic examination alone. We also identified that 10.7% of AMS cases were odontogenic in nature, indicating the need for oral health promotion among people living with HIV, which has been shown to be unsatisfactory [14].

Among patients with OA, about two-thirds were due to ACOM (34.1%) and APOM (31.7%). We did not identify clear reports of their incidence in patients living with HIV, but the possibility of their chronicity in the context of HIV-induced immunosuppression has been frequently reported [15]. Multiple studies report AOE as being common in patients with HIV infection, especially with bacteria such as *Pseudomonas aeruginosa* [15,16,17]. In our analysis, the incidence of AOE was 14.3%, and in one case, *Pseudomonas aeruginosa* was isolated from a female patient who had stage C3 HIV infection.

Due to the retrospective nature of this study, identification of the triggering or the etiological agent was possible in only 35 cases (19.6%). Influenza was the most frequently implicated infection preceding the ENT involvement, and among bacteria, the leading pathogens were *Staphylococcus aureus* (*n* = 6 cases), *Streptococcus pneumoniae* (*n* = 4 cases) and *Haemophilus influenzae* (*n* = 2 cases). Vaccination against type B *Haemophilus influenzae* (Hib) is recommended for patients living with HIV with no previous history of full vaccination and who are associated with other risk factors for developing invasive disease [18]. As seen from the field literature, overall, the risk of invasive disease with Hib is higher in patients with HIV infection than in the general population [18]. Unfortunately, there is no Hib mono-vaccine available in Romania, which makes it difficult to prevent this infection among adults in need and thus among at-risk patients with HIV infection.

These findings taken together reiterate the importance of vaccine prevention for people with HIV infection, especially against influenza, SARS-CoV-2 and pneumococcus. Influenza vaccination is notoriously safe and is associated with acceptable levels of immunogenicity among patients with HIV infection [19,20]. However, in Romania, vaccination rates among this patient population remain generally low, and influenza-related hospitalizations and deaths among patients with HIV infection are reported annually [21,22]. Similarly, SARS-CoV-2 vaccination [23] and pneumococcal vaccination [24] have been shown to be safe and to display acceptable efficacy among patients with HIV infection. However, in order for any vaccine to be efficient, it needs to be administered. Furthermore, while vaccination of the individual provides individual protection, good vaccine coverage is needed for most vaccine-preventable diseases in order to confer herd immunity, or group-level protection. In order to increase the currently suboptimal vaccine coverage levels seen in the general population [25], as well as in the pool of patients living with HIV [26], there is a need to actively involve HIV specialists in promoting vaccination among people living with HIV, explaining the benefits of vaccination, such as reducing the risk of illness, severe complications and hospitalization. Furthermore, tailored intervention strategies should be implemented, including culturally and linguistically appropriate information campaigns, accessible vaccination programs in convenient locations and collaborations with HIV organizations to get the messages about vaccination across to wider groups and to overcome reluctance to vaccinate. It is also crucial to monitor vaccine effectiveness in this population and adjust public health recommendations as new data become available.

In our analysis, we showed that patient sex, HIV infection status or the presence of one or more chronic conditions were not associated with specific types of AS or AO. In contrast, younger age was associated with AMS and ACOM, while AOE was more prominent at older ages. It can be suggested that risk factors or mechanisms contributing to the development, location and type of sinusitis and otitis among HIV patients may be more complex and not directly related to the characteristics highlighted in this study.

In addition, in our analysis, we identified that only 93.3% of patients were on antiretroviral treatment, and 37.0% (27 out of 73 patients for whom viral loads were available) had HIV RNA loads above 1000 copies/mL. These percentages highlight a significant problem in the management of HIV disease, as non-adherence or discontinuation of treatment is a real problem among patients living with HIV. Non-adherence to treatment can lead to multiple negative consequences, including increased viral load, deterioration of the immune system and the development of resistance to antiretroviral drugs. These factors contribute to a worsening of patients’ health status and an increase in HIV-related morbidity and mortality. Moreover, the reasons for patients not following treatment can be varied and complex, including socio-economic factors, stigma associated with the disease, side effects of drugs and lack of psychosocial support. It is essential to identify and address these barriers to improve adherence to treatment. Education and counselling programs, easy access to health services and ongoing support are strategies that can help increase adherence to antiretroviral treatment.

With the changing epidemiological and clinical patterns of infectious diseases following the COVID-19 pandemic [27,28], it is becoming increasingly important to continue to monitor the burden of ENT infections in particular patient populations in order to provide actionable clinical data and to develop algorithms for appropriate management and follow-up, as is already available for other patient populations and other ENT involvements [29].

The retrospective nature of this study is a significant limitation of our report, as this did not allow for the collection of complete data on the exact etiology of the cases studied nor the long-term monitoring of patients’ evolution. These limitations may affect the ability to establish clear causality and to follow the clinical course of the conditions over time. However, to the best of our knowledge, this study represents the first research, both in Romania and globally, that examines a large cohort of more than 3000 patients living with HIV over six years to explore the incidence and characteristics of acute sinus and ear involvement.

## 5. Conclusions

This study determined that hospitalizations due to acute sinus and ear involvement are not isolated events in people living with HIV. The maxillary sinus is frequently involved, and the most common ear manifestations include acute congestive otitis media and acute purulent otitis media. A high degree of immunosuppression, characteristic of advanced C3 stages of HIV infection, serves as a predisposing factor. Prospective monitoring is essential to gain a deeper and more dynamic understanding of how ENT health is affected in individuals with HIV infection. Simultaneously, promoting prevention through vaccination may reduce the burden of ENT infections in this population group.

## Figures and Tables

**Table 1:** Triggers or etiological agents involved in cases of AS and AO.

Etiology	All Cases, *n* (%)	Acute Sinusitis, *n* (%)	Acute Otitis, *n* (%)
	N = 179	N = 149	N = 41
Influenza viruses	16 (8.9)	12 (8.0)	4 (9.8)
SARS-CoV-2	4 (2.2)	4 (2.7)	0 (0.0)
Respiratory syncytial virus	1 (0.6)	1 (0.7)	0 (0.0)
*Streptococcus pneumoniae*	4 (2.2)	3 (2.0)	1 (2.4)
*Staphylococcus aureus*	6 (3.4)	4 (2.7)	2 (4.9)
*Haemophilus influenzae*	2 (1.1)	2 (1.3)	0 (0.0)
*Pseudomonas aeruginosa*	2 (0.6)	1 (0.7)	1 (2.4)

**Table 2:** Clinical and laboratory characteristics of the groups analyzed.

	All Cases, *n* (%)	Only AS, *n* (%)	Only AO, *n* (%)	AS and AO, *n* (%)	*p*-Value
	N = 179	N = 138	N = 30	N = 11	
HIV status					
CD4 level, cells/mm^3^, median (IQR)	508 (246, 761)	486 (247, 761)	483 (69, 805)	694 (489, 809)	0.543
Antiretroviral treatment	171 (95.5)	131 (94.9)	29 (96.6)	11 (100%)	0.868
AIDS stage	120 (67.0)	89 (64.5)	20 (66.6)	11 (100%)	0.802
Clinical characteristics					
Malaise	32 (17.9)	21 (15.2)	7 (23.3)	4 (36.4)	0.147
Fever	118 (65.9)	95 (68.8)	15 (50.0)	8 (72.7)	0.126
Cough	78 (43.6)	63 (45.7)	10 (33.3)	5 (45.5)	0.464
Nasal congestion	114 (63.7)	102 (73.9)	4 (36.4)	8 (26.7)	**<0.001**
Dyspnea	6 (3.4)	5 (3.6)	1 (3.3)	0 (0.0)	0.813
Sore throat	28 (15.6)	20 (14.5)	6 (20.0)	2 (18.2)	0.732
Otalgia	37 (20.7)	0 (0.0)	28 (93.3)	9 (81.8)	**<0.001**
Headache	105 (58.7)	93 (67.4)	6 (20.0)	6 (54.4)	**0.001**
Septum deviation	39 (21.8)	34 (24.6)	1 (3.3)	4 (36.4)	**0.018**
Turbinate hypertrophy	40 (22.3)	36 (26.1)	1 (3.3)	3 (27.3)	**0.023**
Blood counts					
Increased WBC	91 (50.8)	64 (46.4)	21 (70.0)	6 (54.5)	**0.009**
Increased neutrophils	86 (48.0)	62 (44.9)	18 (60.0)	6 (54.5)	0.097
Increased lymphocytes	5 (2.8)	1 (0.7)	4 (13.3)	0 (0.0)	NA
Decreased lymphocytes	64 (35.8)	51 (37.0)	10 (33.3)	3 (27.3)	0.776
Increased monocytes	3 (1.7)	2 (1.4)	1 (3.3)	0 (0.0)	NA
Inflammatory syndrome					
Increased CRP	133 (74.3)	99 (71.7)	25 (83.3)	9 (81.8)	0.051

AS—acute sinusitis, AO—acute otitis, WBC—white blood cells, CRP—C reactive protein, ESR—erythrocyte sedimentation rate, and NA—not applicable. In bold are highlighted comparisons with statistical significance.

## Data Availability

The data are available upon reasonable request to the corresponding author.
